# Strategic site selection for placement of HIV early infant diagnosis point-of-care technology within a national diagnostic network in Lesotho

**DOI:** 10.4102/ajlm.v10i1.1156

**Published:** 2021-08-24

**Authors:** Anafi Mataka, Esther A.J. Tumbare, Tsietso Motsoane, David Holtzman, Monkoe Leqheka, Kolisang Phatsoane, Emma Sacks, Anthony Isavwa, Appolinaire Tiam

**Affiliations:** 1 African Society for Laboratory Medicine, Addis Ababa, Ethiopia; 2 Elizabeth Glaser Pediatric AIDS Foundation (EGPAF), Maseru, Lesotho; 3 Laboratory Services, Ministry of Health, Maseru, Lesotho; 4 Partners in Health, Maseru, Lesotho; 5 School of Public Health, George Washington University, Washington, District of Columbia, United States; 6 Elizabeth Glaser Pediatric AIDS Foundation (EGPAF), Nairobi, Kenya; 7 Elizabeth Glaser Pediatric AIDS Foundation (EGPAF), Washington, District of Columbia, United States

**Keywords:** HIV early infant diagnosis, point-of-care, increased health access, site selection

## Abstract

**Background:**

New technologies for rapid point-of-care (POC) diagnostic tests hold great potential for improving the health outcomes of HIV-exposed infants. POC testing for HIV early infant diagnosis (EID) was introduced in Lesotho in late 2016. Here we highlight critical requirements for selecting routine POC EID sites to ensure a sustainable and optimised EID diagnostic network.

**Intervention:**

Lesotho introduced POC EID in a phased approach that included assessments of national databases to identify sites with high test volumes, the creation of local networks of sites to potentially increase access to POC EID, and a standardised capacity assessment to determine site readiness. Potential site networks comprising ‘hub’ testing sites and ‘spoke’ specimen referring sites were created.

**Lessons learnt:**

After determining optimal placement, a total of 29 testing facilities were selected for placement of POC EID to potentially increase access to 189 facilities through the use of a hub-and-spoke model. Site capacity assessments identified vital human resources and infrastructure capacity gaps that needed to be addressed before introducing POC EID and informed appropriate POC platform selection.

**Recommendations:**

POC placement involves more than just purchasing the testing platforms. Considering the relatively small proportion of sites that can be eligible for placement of a POC platform, utilising a hub-and-spoke model can maximise the number of health facilities served by a POC platform while reducing the necessary capacity building and infrastructure investments to fewer sites.

## Background

In Lesotho, a country with a high burden of HIV infection of nearly one in every four pregnant women and adults aged between 15 years and 49 years,^1^ antenatal care and HIV treatment for women and children are delivered at three levels: primary, secondary and tertiary. At the primary healthcare level are health centres, health posts and all community-level outreaches. District hospitals serve as next level referral facilities for all health centres in the district and refer cases to a national referral hospital at the tertiary level. Laboratory services are structured similarly. However, in 2016 only one laboratory, the National Reference Laboratory (NRL) in the capital city, processed dried blood spot (DBS) samples for HIV early infant diagnosis (EID) testing, and this was done using conventional complex laboratory equipment. The DBS samples were transported from various health facilities all over the country to the NRL using a motorcycle-based national sample transport system. One of the main challenges with this centralised EID system was long turn-around times (TATs) – typically between 30 days and 90 days – for returning results to local health centres and caregivers.^2,3^

Reasons for the long TATs include delays at the clinics before specimens are dispatched to the district hospital laboratory and delays in sending samples from the district hospital laboratory to the NRL. Also, at the NRL, waiting to batch samples before analysis to allow sufficient numbers for full test runs on the instruments added to the long TAT. Further delays occur during the return of paper-based results to clinics through the same sample transport system before finally reaching caregivers, who may not have telephone access to facilitate quicker result communication or live far from facilities. To strengthen this centralised system, the Ministry of Health has implemented mechanisms such as routine training on DBS collection to ensure the quality of specimens and reduce rejection rates and provision of more motorcycles to increase the pick-up frequency of DBS and other clinical samples from facilities; however, many challenges remain.

New technologies for rapid, point-of-care (POC) molecular diagnostic tests hold great potential for improving timely management of infants with HIV infection by eliminating delays in the return of results and enabling rapid initiation of antiretroviral therapy.^4,5^ Lesotho embraced POC EID testing in 2016, making it one of the first countries to introduce the technology into routine clinical settings. The POC platforms were initially thought to be particularly useful for hard-to-reach areas, where delays in result return were often the longest. However, given the large numbers of primary and secondary health facilities (255 such facilities in Lesotho) and the limited number of platforms that were available due to funding constraints, not all health facilities or service delivery points could receive POC EID platforms. Hence, strategic placement of the POC EID platforms was necessary.

Before 2016, POC EID testing was primarily introduced in controlled environments such as research and pilot projects in sub-Saharan Africa.^5,6,7^ Many POC platforms for various diagnostic tests have been deployed in healthcare settings. However, few implementers have tried to describe and explain the actual real-life process for introducing and integrating a new POC into a national health system for routine use. A better understanding of how the introduction of new POC technologies is implemented could help us anticipate possible challenges and identify elements needed for successful implementation in other limited-resource settings. This article aims to share Lesotho’s experiences of a systematic approach to identifying and assessing the readiness of potential POC EID sites and to highlight the critical requirements for selecting sites to integrate routine POC EID into a sustainable and optimised EID diagnostic network.

## Description of the intervention

### Ethical considerations

The approval to conduct site selection and capacity assessments was obtained from the Lesotho Ministry of Health Research and Ethics Committee (Approval number ID 29-2016, dated 13 January 2016).

### Setting

The processes of selecting sites eligible for POC EID testing, assessing site capacity and needs, and designing the POC EID network were carried out for all the ten districts of Lesotho in the year 2016. The geography of Lesotho’s highly mountainous kingdom poses many challenges to providing high-quality healthcare because of the difficulties to reach the remote facilities, especially those high up the mountains.

The site selection was carried out in two main phases: an initial desk analysis to determine potential sites that could access POC EID and capacity assessments to determine the readiness for implementation of POC testing.

### Desk analysis to determine potential health facilities eligible for POC EID

Two data sources were used in the desk analysis, namely the 2014 national population projections (unpublished) for catchment areas of health facilities (*n* = 158) in rural, peri-urban and urban settings and the Ministry of Health’s laboratory information system.

Data on the expected volume of EID tests in each site were extracted; these data were based on the highest number of expected pregnancies from HIV-positive women, the expected number of HIV-exposed infants, or historical DNA polymerase chain reaction tests. Data was also collected on the availability of onsite antiretroviral therapy or paediatric antiretroviral therapy services, facilitating the ability to use results immediately in a real POC setup.

The Ministry of Health’s laboratory information system was queried in March 2016 for all EID test samples collected from HIV-exposed infants in the 255 facilities nationwide in the previous year (January 2015 to December 2015). The data were disaggregated to total samples per site in a year. To get testing rates per day, the annual test volume was divided by 12 months, following which the result was divided by 22, which is the number of working days per month.

A threshold of 0.5 tests per day (at least one test every two days) was set as the minimum test volume for sites that would receive a POC platform. This threshold was regarded as sufficient to maintain operator competency and provided a throughput of tests high enough to justify the cost of the POC platforms. Using a hub-and-spoke model, several health facilities with low test volumes were combined with other low-volume sites or with sites with higher test volumes to form local testing networks that reached or exceeded the threshold of 0.5 tests per day. In this model, the sites were grouped around a central location (hub) with a testing platform that would serve the surrounding health facilities (spokes). Criteria for eligibility of a spoke site included proximity to the nearest potential POC hub site and the existence of a sample transport system to a nearby possible hub site. Proximity was defined as facilities that were, on average, no further than 60 km from the possible testing site. Spoke sites are required to send samples using sample transport carriers to a nearby hub site that provides onsite POC EID testing service, thus eligibility was also based on the ability to send samples in ethylenediaminetetraacetic acid-treated capillary tubes according to laboratory and manufacturers’ standards. The latter recommends specimens to be carried within 24 h before testing if kept at ambient temperature and within three days if kept and transported between 2 °C and 8 °C. These hubs and spokes already existed as part of the national sample transport system; hence, no additional transport or new routes were created.

Eligible sites were then scheduled for site capacity assessments to determine their readiness for POC EID introduction. The first 15 sites assessed were located in rural, peri-urban and urban settings across all the 10 districts. The remaining 14 testing sites were also scheduled to undergo capacity assessments later and were placed in a ‘scale-up’ phase of the implementation.

### Site capacity assessments of selected POC EID sites

Capacity assessments were conducted using a standardised checklist adapted from the Stepwise Process for Improving the Quality of HIV-Related Point-of-Care Testing checklist version 2.0, 16 September 2014.^8^ The Stepwise Process for Improving the Quality of HIV-Related Point-of-Care Testing checklist attempts to harmonise with international regulations to evaluate sites consistently. The adapted tool has eight domains and 49 questions. Each domain had questions on how well the facility performs specific tasks. Based on the findings, each of the eight domains was given a weighted score. The total for all the domains was computed to arrive at a numerical percentage score. Site readiness level, ranging from Level 0 to Level 4, was determined based on these scores. Level 0 (0% – 40%) sites were those that needed improvement in all areas, Level 1 (40% – 59%) sites required improvement in specific areas, while Level 2 (60% – 79%) sites were partially eligible. Level 3 (80% – 89%) sites were close to pilot site capacity but needed some upgrades or improvements, while Level 4 (90% or higher) sites were fully eligible for selection as pilot sites.

### Data management and analysis

Descriptive analyses (percentage scores by checklist domain for each health facility and median scores per checklist domain) were carried out using Microsoft Excel (Microsoft Corporation, Redmond, Washington, United States). Statistical comparisons between facilities that underwent capacity assessments were not conducted due to the small sample size (*n* = 15). Data on historical laboratory EID test volumes were exported into an Excel database to calculate the test rate per site and aggregated test volumes for each potential local testing network.

## Lessons learnt

This article presents the steps taken before the introduction of POC platforms in Lesotho, including health facility pre-selection, data analysis aimed at maximising the use of available POC platforms, and the determination of site readiness for POC EID.

### Desk analysis to increase access to POC EID testing

Important considerations for setting up POC EID testing at a health facility include sufficient test volumes of at least one test every two days and the availability of HIV treatment services or referral treatment, including paediatrics, at the health facility or within a set distance to allow quick referrals. The geographic locations are also an important consideration as TATs for delivery of EID results from the centralised laboratory may be prolonged in hard-to-reach areas and affect the ability to monitor the performance of EID testing.

Most of the sites assessed had test volumes that were too low to justify the placement of an instrument. Creating local testing networks to increase testing volumes can improve access in underserved areas that are very far from the NRL. We considered all facilities with records of having collected samples for polymerase chain reaction from infants in the previous year. It has been demonstrated that some areas in hard-to-reach regions have challenges sending samples to the national laboratories and often have very long TATs from sample collection to return of results.^3^ Thus, in developing an optimised network map for both conventional and POC EID testing, POC testing should be considered not only as a tool for hard-to-reach areas but also to reduce TAT from sample collection to return of results. The availability of systems for sample transport to the hubs with POC platforms would mean that although these sites would not get the EID result on the same day, they would get them in a few days since most of the sites were serviced by the sample transport system at least twice a week. This would be a potentially significant improvement compared to conventional EID when specimens are processed in the conventional laboratory, where results often took quite long (usually months) to reach the caregiver for several reasons.^3^ These POC sites would also take some of the burden off conventional laboratories that are overloaded with viral load (VL) testing and improve TAT.

Following the desk analysis, in which a total of 255 sites were analysed, POC EID was determined to be potentially suitable for introduction into 29 testing sites. Through the use of the hub-and-spoke model, it was found that access to POC EID could be increased 6.5-fold from 29 to 189 sites; five of the 29 sites were stand-alone testing sites with no associated spoke sites and 24 were testing hubs that were designated to receive samples from an additional 160 spoke sites (Table 1). Thus, in total, 189 sites were selected for possible access to POC EID based on the hub-and-spoke model, most (88%; 166/189) of which had fewer than one test every two days (< 0.5 EID/day). The remaining 66 of the 255 sites would continue to be served by the conventional system because of their proximity to the NRL and the sample transport network.

**TABLE 1:** Outcomes of the desk analysis for site selection and mapping for placement of HIV point-of-care early infant diagnosis using a hub-and-spoke model in Lesotho, 2016.

Outcome of site selection analysis	Number	%
Total number of sites analysed	255	-
Number of districts covered	10	100
Number of sites selected for access to POC EID on hub-and-spoke model	189	74
Number of sites with an average demand of ≥ 0.5 EID/day	23	12
Number of sites with an average demand of < 0.5 EID/day	166	88
Median historical annual EID demand per site [range]	33 EID/year [1–624]	-
Median theoretical annual EID demand per site based on HIV-exposed infants	62 EID/year (0–616)	-
Number of sites (stand-alone or hub) potentially eligible for POC EID testing (≥ 0.5 EID/day and offering paediatric antiretroviral therapy)	29	-
Number of stand-alone sites	5	-
Number of possible hub sites (from networking non-eligible low demand sites)	24	-

EID, early infant diagnosis; POC, point-of-care.

During the assessment of each spoke site, it was essential to assess distance and accessibility to the nearby potential testing site in terms of how samples could be transported in ethylenediaminetetraacetic acid-treated capillary tubes according to laboratory and manufacturers’ standards. For the optimal quality of samples, the facility should be within 60 min of the hub site by normal transport modes. Where this is not possible, a DBS sample becomes ideal; this is in place in many countries. However, the challenge of long TATs for results delivery still exists. Therefore, governments need to strengthen systems to improve the value of using alternative sample types for EID and VL.^9^ Creating a local hub-and-spoke network contributes to strengthening the overall network by potentially reducing TAT and placing less burden on the conventional system that could use the space for VL testing. Due to the high workload at the NRL, which had a backlog, EID was run only 1–2 days per week.

The platforms selected for potential POC facilities, namely the Cepheid GeneXpert (GX-VI) four-module instrument (Cepheid, Sunnyvale, California, United States) and the m-Pima (Abbott Laboratories, Chicago, Illinois, United States), formerly the Alere q, are capable of conducting other tests beyond HIV EID. For example, both can do HIV VL testing, while the Cepheid GeneXpert can also test for other infections such as tuberculosis, hepatitis, human papillomavirus and more.^10^ However, at the time of selection, there was no evidence of the feasibility of POC testing for HIV VL monitoring. Since these were new, it was decided to start testing for EID and not perform integrated testing.

### Point-of-care site capacity assessments

**FIGURE 1:**
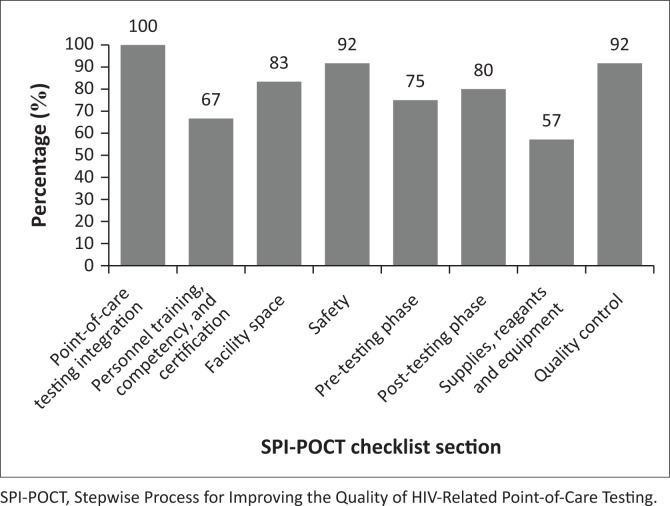
Median performance scores per Stepwise Process for Improving the Quality of HIV-Related Point-of-Care Testing checklist domain of potential point-of-care early infant diagnosis health facilities (N = 15) in Lesotho, February 2016.

A total of 15 sites with the highest test volumes were assessed for capacity to conduct POC EID testing. Of the 15 facilities assessed, only one facility was fully eligible (Level 4) for implementation of POC EID (Table 2). The majority (*n* = 14) were not immediately ready to implement new POC EID testing and required structural upgrades, including process-related improvements. The required infrastructural improvements included providing room and tables for POC platforms and shelves to store POC commodities, providing fridge thermometers, and installing air conditioners and power inverters or backup for some sites. Required process-related improvements included improving site-level stock management through training, integrating standardised forms to document patient and specimen information, as well as printing and distributing EID testing algorithms, job aids, logbooks, and POC EID testing forms. Documentation of standard operating procedures, safety practices and data management mechanisms also needed to be improved in all facilities (Table 3). Also, competent POC platform operators were required to be present or recruited in all facilities. Across all health facilities, the highest median scores were obtained in integrating POC services into HIV care (100%), which evaluated the prior experience in POC testing for any disease, such as rapid testing for HIV and malaria. This was followed by the domains on quality control (92%) and safety practices (92%). The lowest median scores were obtained in the supplies, reagents and equipment management domain (57%) and the personnel training, competency and certification domain (67%) (Figure 1).

**TABLE 2:** Readiness of selected health facilities in Lesotho (*N* = 15) in 2016 to introduce HIV point-of-care early infant diagnosis based on a standardised tool adapted from the Stepwise Process for Improving the Quality of HIV-Related Point-of-Care Testing checklist version 2.0. 9/16/2014.

Type of facility	Level 0†	Level 1‡	Level 2§	Level 3¶	Level 4††	Total
District hospital	0	0	5	5	1	11
Health centre	0	0	2	2	0	4
All facilities	0	0	7	7	1	15

*Source*: Adapted from the Stepwise Process for Improving the Quality of HIV-Related Point-of-Care Testing (SPI-POCT) checklist version 2.0, 9/16/2014.^8^

† , 0% – 40% (needs improvement in all areas);

‡ , 40% – 59% (needs improvement in specific areas);

§ , 60% – 79% (partially eligible; needs upgrades/improvements);

¶ , 80% – 89% (close to pilot site capacity; needs some upgrades/improvements);

†† , 90% or higher (fully eligible for selection as a pilot site).

**TABLE 3:** Common gaps identified during the site capacity assessments of health facilities (*N* = 15) in Lesotho, 2016.

Key gap	Number of facilities
Lack of designated physical spaces for POC testing, including safe and secure storage space.	9
Lack of a policy specifying which cadres may perform POC testing.	8
Not all personnel certified competent for POC testing.	12
No or inadequate documentation of work instructions and standard operating procedures for the pre-testing and testing phases.	10
Inadequate quality of test monitoring and POC testing supervision.	11
Poor information and data management.	4
No documented procedures for stock and supply chain management.	8
Irregular monthly inventory counts for all supplies and reagents.	6
No documented training on handling biohazardous material and workplace safety.	7
Lack of experience with routine maintenance and troubleshooting or repair of POC equipment and instruments.	5
Poor or no temperature monitoring at the designated or potential POC area and reagent storage space.	9

POC, point-of-care.

There was adequate space in all the 15 facilities where POC testing equipment could be placed, although not all were designated for POC. All facilities had reliable paediatric HIV counselling and treatment services at the facility or within a reasonable distance. However, despite the prior experience of conducting POC tests for rapid HIV or any other disease, there were several common gaps across all health facilities. Several sites had inadequate security or lacked room temperature monitoring at the POC testing or storage areas for commodities. The facilities also had poor documentation, did not sufficiently manage the supplies of already existing POC tests and lacked storage capacity for POC commodities (Table 3).

Before the introduction of POC EID in these sites, room temperature monitoring thermometers, security padlocks, tables, and cabinets to store POC commodities had to be procured. In addition to equipment-related training, all POC testing personnel were trained on supplies and commodities management, safety, pre-testing, and post-testing procedures, including result management and POC EID use in the existing national EID algorithm. Since POC EID was new in the country, a testing algorithm for use at POC sites was developed and provided to facilities as a reference guide. The algorithm followed the same testing interval as the existing national algorithm. Still, it specified how to handle test results, given that tests are available on the same day, which was not the case with the current centralised system.

Another critical consideration was the duration of the steps taken to introduce POC testing. The site selection process took three months, while the procurement, including site upgrades, took nearly four months to complete. It is therefore essential to take into account the time lag, including procurement lead times for commodities. Where possible, it is best to consider procuring materials for upgrades locally to reduce lead times associated with external procurement.

The procurement of POC EID platforms was planned to suit each site’s needs-based infrastructure, availability of reliable electricity, dynamics of daily patient testing volumes, and other characteristics as determined on the findings of the physical capacity assessments. The selection was informed by a side-by-side analysis, using a tool^12^ that compared three platforms based on various characteristics such as maximum throughput, storage temperature of reagents, power requirements, type of sample (whole blood or capillary), time to result, waste management needs and regulatory approvals. Many of these elements were assessed during the capacity assessment. The Cepheid GeneXpert four-module instrument and a laptop computer were placed in 16 sites, and the m-Pima, formerly the Alere q, was found to be suitable for 13 of the sites. A phased rollout plan was developed, which involved the implementation of five sites (three hubs and two stand-alone sites) in the first phase, the implementation of a further ten sites after 6 months with the modification of appropriate steps based on lessons learned in the first phase, and the implementation of 14 additional sites in a final scale-up phase after 12 months. At the time of writing, a total of 23 hubs with 131 spokes and six stand-alone sites had been implemented.

We used historical testing rates to estimate expected test volumes. One limitation of this method is that we could not fully guarantee the same test volumes in future because other variables such as population movements and birth rates could change. While the results of the site selection and network mapping process showed that POC EID could potentially increase access to testing, a post evaluation is needed to verify this potential impact in this setting. Recent data from the multi-country evaluation showed that POC EID resulted in better outcomes for HIV-exposed and HIV-positive infants. The data, which included Lesotho, showed that compared to conventional laboratory testing,^12^ there was a five-fold increase in the proportion of infants receiving EID results within 30 days of the EID test and the number of infants initiated on antiretroviral therapy within 2 months more than doubled.

Given the many steps involved in introducing POC testing in a country and the data requirements to make evidence-based decisions, all key stakeholders, must be identified and consulted. Stakeholders’ participation should be coordinated, preferably by the directorate of laboratories or equivalent leadership for diagnostics in a country. Such stakeholders include development partners who may also have plans to roll out POC in specific areas, other ministries such as the information ministry to advise on data connectivity, and environmental units to guide waste management. Clinicians and key technical working groups such as the Prevention of Mother to Child Transmission and the Laboratory Technical Working Groups are also important stakeholders in their advisory roles for the Ministry of Health. Supply chain and procurement units need to be looped in early to avoid unnecessary delays for procured materials.

## Recommendations

Our site selection process suggests that placement of POC EID platforms at well-prepared sites within diagnostic networks in limited-resource settings can improve access and that optimal introduction of POC EID transcends mere POC platform procurement. For pre-selected potential sites meeting all preliminary criteria, more detailed site capacity assessments of human resources and infrastructure capacity are required. In the case of many sites being ineligible for a POC platform based on insufficient testing volumes, and considering that the vast majority of them require some level of capacity building or infrastructure upgrade, a hub-and-spoke model can be adopted to potentially increase access to POC EID.
